# Psoralen and Isopsoralen Activate Nuclear Factor Erythroid 2‐Related Factor 2 Through Interaction With Kelch‐Like ECH‐Associated Protein 1

**DOI:** 10.1002/fsn3.4768

**Published:** 2025-01-24

**Authors:** Chengyu Lv, Song Wang, Chang Sun, Jing Liu, Yihao Chen, Chao Wang, Cuiping Yuan, Fengxian Qin, Tiezhu Li

**Affiliations:** ^1^ Institute of Agro‐Food Technology Jilin Academy of Agricultural Sciences (Northeast Agricultural Research Center of China) Changchun China; ^2^ Jilin Ginseng Academy Changchun University of Chinese Medicine Changchun China

**Keywords:** antioxidant response element, isopsoralen, molecular docking, Nrf2, psoralen

## Abstract

As natural furocoumarins, psoralen and its isomer isopsoralen are widely distributed in various fruits including *Ficus carica* L., vegetables including celery, and medicinal herbs including *Psoralea corylifolia* L. Although psoralen and isopsoralen have been used as dietary supplements because of their bioactivities such as antibacterial and anti‐inflammatory properties; however, the potential mechanisms underlying the antioxidant activities of these two furocoumarins still need to be explored. Hence, the aims of this work were to examine the activation of nuclear factor erythroid 2‐related factor 2 (Nrf2) by psoralen and isopsoralen, as well as the binding interaction of Kelch‐like ECH‐associated protein 1 (Keap1) with these two furocoumarins. Interestingly, both psoralen and isopsoralen induced Nrf2 nuclear translocation in a dose‐dependent manner in HEK293T cells. These two furanocoumarins also activated antioxidant response element (ARE)‐driven luciferase activity. The mRNA expression of GCLM, HO‐1, and NQO1 genes was significantly upregulated by treatment of HEK293T cells with psoralen and isopsoralen, respectively. Similarly, the expression of proteins can be promoted. Both psoralen and isopsoralen were located in the top of the central pocket of the Keap1 Kelch domain, suggesting that they were natural ligands of Keap1. In conclusion, both psoralen and isopsoralen activate Nrf2 through interaction with Keap1, thereby serving as natural antioxidants.

## Graphical abstract

Psoralen and its isomer isopsoralen induced the gene and protein expression of Nrf2‐mediated antioxidant targets. Psoralen and isopsoralen activate Nrf2 through interaction with Keap1. The molecular basis for the binding of psoralen and isopsoralen to Keap1 is explored.

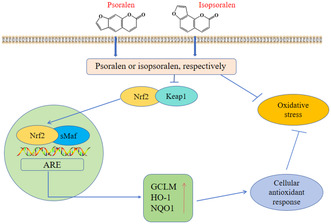

## 1 Introduction

Nuclear factor erythroid 2‐related factor 2 (Nrf2) is a member of the Cap'N'Collar family and is a critical transcription factor in the regulation of oxidative stress (Luo et al. 2023; Tu et al. 2019). Normally, Nrf2 forms a dimer with the inhibitory protein Kelch‐like ECH‐associated protein 1 (Keap‐1) that binds to and anchors actin in the cytoplasm of the cells, which is rapidly disassembled via the ubiquitin proteasomal pathway (Ding, Zhao, and Chen 2022; Taguchi, Motohashi, and Yamamoto 2011). When cells are stimulated by oxidative stress, Keap‐1 conformational changes inhibit Nrf2 ubiquitination, and Keap‐1/Nrf2 dimer uncoupling occurs (Serafini et al. 2019; Zhang et al. 2017). Nrf2 translocates into the nucleus and recognizes antioxidant response elements (ARE), thereby initiating the transcription of downstream antioxidant stress enzymes and phase II detoxification enzyme genes (Ding, Zhao, and Chen 2022; Liu et al. 2017; Tu et al. 2019).

The Nrf2‐ARE pathway plays a key role in maintaining cellular redox homeostasis and can be induced by both exogenous stimuli (superoxide, metal ions, electrophilic reagents) and endogenous stimuli (reactive oxygen radicals). Interestingly, natural small‐molecule compounds can interact with Keap1 protein to activate the Nrf2‐ARE pathway and modulate the expression of glutamate‐cysteine ligase (GCLM), heme oxygenase 1 (HO‐1), nicotinamide adenine dinucleotide phosphate (NADPH) quinone dehydrogenase 1 (NQO1), exerting their antioxidant effect (Eggler, Gay, and Mesecar 2010; Tonolo et al. 2020; Zou et al. 2021). Pterostilbene protects HaCaT cells from UVB‐induced photodamage by activating the PI3K pathway, promoting Nrf2 nuclear translocation and inducing the expression of antioxidant enzymes.

Psoraleae Fructus (PF) is the dried ripe fruit of *Psoralea corylifolia* L., family Leguminosae, and is known as “Buguzhi” in China (Ren et al. 2020; Wang et al. 2023). PF is recorded in “Leigong's Treatise” and is widely used as a Chinese herbal medicine. According to the Chinese pharmacopeia, it can be used to nourish the kidneys and tonic yang, improve blood circulation, strengthen the spleen and stomach, and promote inspiration and check diarrhea (Xin et al. 2010; Yang et al. 2018). In modern pharmacological studies, a variety of functional components have been isolated from PF, including coumarins, flavonoids, monoterpene phenols, and benzofurans (Li, Zhou, Zheng et al. 2019; Li, Huang, Jie et al. 2019). They are commonly used to treat osteoporosis, inflammation, cancer, vitiligo, psoriasis, and eczema (Hsieh et al. 2014; Lee et al. 2015; Zhang et al. 2022).

As natural furocoumarins, psoralen and its isomer isopsoralen are widely distributed in various fruits including *Ficus carica* L., vegetables including celery, and medicinal herbs including *Psoralea corylifolia* L. (Sui et al. 2020). Psoralen and isopsoralen have been used as dietary supplements because of their bioactivities such as antibacterial and anti‐inflammatory properties (Hai et al. 2017; Shin, Shon, and Youn 2013). Psoralen and isopsoralen have previously been used to treat rheumatoid arthritis in a mouse model (Han et al. 2021). Moreover, psoralen and isopsoralen exerted antifibrotic and antiapoptotic effects on streptozotocin‐induced diabetic mice, thereby reducing glomerular thylakoid cell injury and attenuating diabetic nephropathy (Seo et al. 2017). To sum up, the potential mechanisms for the antioxidant activities of psoralen and isopsoralen still need to be explored.

**FIGURE 1:**
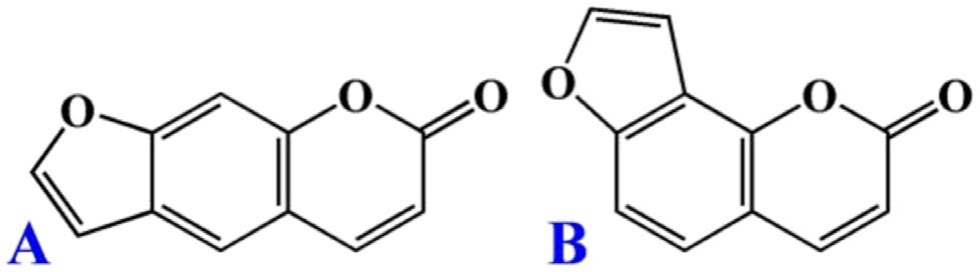
The structures of psoralen (A) and isopsoralen (B).

This work aims to investigate the activation of Nrf2 by psoralen and isopsoralen (Figure 1), as well as the binding interaction of Keap1 with these two furocoumarins. The in vitro antioxidant capacity of psoralen and isopsoralen was determined by free radical scavenging assay. The cytotoxicities of psoralen and isopsoralen against HEK293T cells were examined by MTT assay, and then their induction of Nrf2 nuclear translocation was determined by a Western blotting assay. The transcriptional activation of ARE‐driven reporter activities of psoralen and isopsoralen was further assessed by a reporter gene assay. The effects of psoralen and isopsoralen on mRNA and protein expression levels, including GCLM, HO‐1, and NQO1, were investigated. In addition, the binding mode and binding stability of Keap1 with these two furocoumarins were explored by molecular docking.

## 2 Materials and Methods

### 2.1 Chemicals and Materials

Psoralen and isopsoralen were obtained from CATO Research Chemicals Inc. (Guangzhou, China). *tert*‐Butylhydroquinone was purchased from MedChemExpress (Shanghai, China). 3‐(4,5‐Dimethylth‐iazol‐2‐yl)‐2,5‐diphenyltetrazolium bromide (MTT) was purchased from Genview (TX, USA). Dimethyl sulfoxide (DMSO) was purchased from MP Biomedicals (California, CA). Dulbecco's modified Eagle medium (DMEM) was obtained from Wuhan Pricella Biotechnology Co. Ltd. (Wuhan, China). Fetal bovine serum (FBS) was obtained from OriCell (Guangzhou) Biotechnology Co., (Guangzhou, China). Anti‐GCLM, anti‐HO‐1, anti‐NQO1, anti‐LaminB1, anti‐Nrf2, and anti‐GAPDH were purchased from Proteintech (Wuhan, China). Dual‐Glo Luciferase Reporter Assay System and ECL Western Blotting Substrate were purchased from Promega (Madison, WI, USA).

### 2.2 DPPH Radical‐Scavenging Assays

One hundred microliters of psoralen or isopsoralen at concentrations of 0.01, 0.08, 0.1, 0.8, 1, 8, and 10 mM was mixed with 100 μL of freshly prepared DPPH solution (80 mg/L). The mixture was reacted in the dark for 30 min at room temperature. The absorbance values were determined at a wavelength of 517 nm.

### 2.3 ABTS Radical‐Scavenging Assays

ABTS^•+^ working solution was prepared from a 7 mM ABTS solution and a 2.5 mM potassium persulfate solution, and the absorbance value was adjusted at 734 nm to 0.70 ± 0.02. Four hundred microliters of psoralen or isopsoralen at concentrations of 0.01, 0.08, 0.1, 0.8, 1, 8, and 10 mM was mixed with the ABTS^•+^ solution, respectively. After 30 s of vigorous shaking, the mixtures were made to react for 6 min in the dark. The absorbance values were determined at a wavelength of 734 nm.

### 2.4 Hydroxyl Radical‐Scavenging Assays

Different concentrations (0.01, 0.08, 0.1, 0.8, 1, 8, and 10 mM) of psoralen or isopsoralen were mixed with 9 mM ferrous sulfate and 10 mM hydrogen peroxide. They were reacted in the dark (37°C) for 1 h and mixed with 9 mM salicylic acid. They were then reacted in the dark (37°C) for 30 min. The absorbance values were determined at a wavelength of 510 nm.

### 2.5 Cell Culture and Cell Viability

Human embryonic kidney (HEK293T) cell line was purchased from Wuhan Sunncell Biotechnology Co. (Wuhan, China). The cells were inoculated in DMEM medium containing 10% (v/v) FBS and were incubated at 37°C with 5% CO_2_ under 90% humidity. Ten thousand HEK293T cells per well were inoculated in 96‐well plates, with 100 μL DMEM medium added, and incubated for 24 h. Different concentration gradients of psoralen, isopsoralen, and H_2_O_2_ were added. After 24 h of exposure, the supernatant was discarded. The MTT was solubilized in PBS to a final concentration of 5 mg/mL. Ten microliters of MTT solution was added per well and incubated at 37°C with 5% CO_2_ under 90% humidity for 4 h. The entire supernatant was discarded, and 150 μL DMSO was added to dissolve formazan. A microplate reader was used to measure the OD values at 570 nm.

### 2.6 ARE‐Driven Luciferase Reporter Gene Assay

HEK293T cells were inoculated in 24‐well cell culture plates in 500 μL phenol red‐free DMEM medium and cultured for 24 h. The reporter plasmid pARE‐Luc, containing AREs, and the internal plasmid pRL‐SV40, expressing Renilla luciferase, were used for reporter gene detection. HEK293T cells were transiently transfected with Lipofectamine 2000 at a ratio of 8:1 for 4 h. The compounds to be tested, *tert*‐butylhydroquinone, psoralen, or isopsoralen, were first added and then exposed for 24 h. Subsequently, passive lysis buffer was used to lyse the treated HEK293T cells and to measure the luciferase activity of the cell lysates on a microplate reader using the Dual‐Glo Luciferase Reporter Assay System.

### 2.7 Gene Expression Assay

HEK293T cells were incubated in 6‐well plates with 2 mL of DMEM for approximately 24 h, and exposed to concentrations of 10, 20, and 40 μM psoralen and isopsoralen for 18 h. The cells were treated with H_2_O_2_ for another 8 h. Trizol reagent was used to extract RNA from the cells, and the obtained RNA was reverse transcribed into cDNA. The primer sequences used for RT‐PCR have been described in previous studies (Ma et al. 2022). The RT‐qPCR was performed using the LightCycler 96 thermocycler. *GAPDH* was adopted as the reference gene. The results were normalized to the DMSO treatment and the relative gene expression was calculated using the 2^−∆∆C^_t_ method.

### 2.8 Western Blot Analysis

HEK293T cells were seeded in 10 cm disks (5 × 10^6^ cells per disk) and cultured for 24 h. The cells were collected using RIPA buffer containing protease/phosphatase inhibitors. The proteins in the cells were extracted, and their concentrations were standardized. HEK293T cell proteins were separated by SDS‐PAGE electrophoresis and then transferred to PVDF membranes (GE Healthcare, USA). A 5% bovine serum albumin (BSA) was used to block the PVDF membrane, and diluted primary antibodies (including anti‐GCLM, anti‐HO‐1, anti‐NQO1, anti‐Nrf2, anti‐LaminB, and anti‐GAPDH) were incubated at 4°C overnight. The next day, the membranes were washed with TBST solution and then incubated with horseradish peroxidase–conjugated secondary antibody for 1 h at room temperature protected from light. The ECL immunoblotting assay kit detected protein bands.

### 2.9 Molecular Docking of Keap1 With Psoralen and Isopsoralen

The binding mode of Keap1 with psoralen and isopsoralen was explored by molecular docking in this work. The co‐crystal structure of the Kelch domain of human Keap1 in the presence of (*S,R,S*)‐1a was obtained from PDB with the ID of 4L7B (Jnoff et al. 2014). The co‐crystal ligand was removed by Chimera in order to prepare the protein structure of the Keap1 Kelch domain in AutoDockTools‐1.5.6. The structures of these two furocoumarins were produced by GaussView 5.0 and optimized by Gaussian 09W. After the verification of docking calculation, each furocoumarin was docked to the active site of the Keap1 Kelch domain. Subsequently, the detailed binding mode of Keap1 with these two furocoumarins was visualized in PyMOL.

### 2.10 Statistical Analysis

All experiments were conducted three times independently, and the results were expressed as the mean ± standard deviation (SD). Tukey's multiple comparison test was performed to compare the two groups.

## 3 Results and Discussion

### 3.1 Free Radical Scavenging Ability of Psoralen and Isopsoralen

**FIGURE 2:**
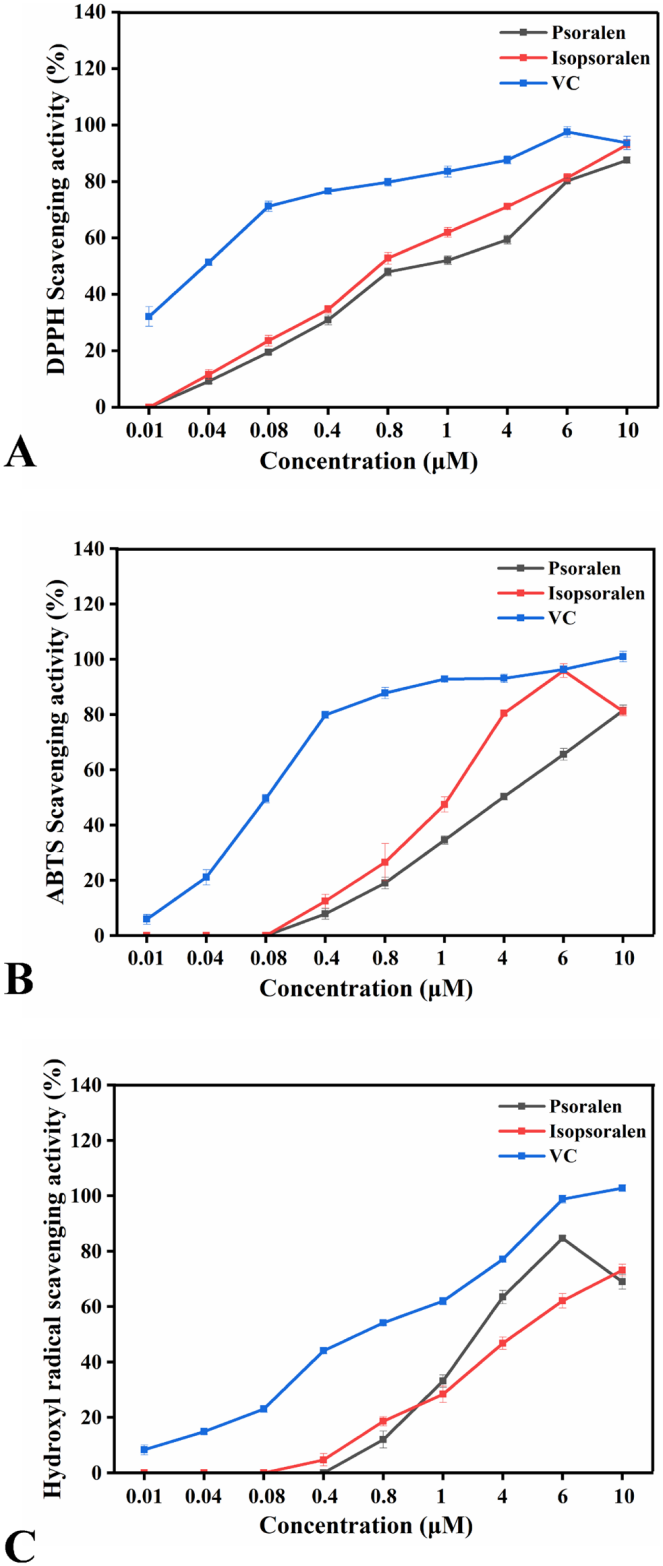
Free radical scavenging ability of psoralen and isopsoralen. (A) DPPH radical scavenging ability. (B) ABTS radical scavenging ability. (C) Hydroxyl radical scavenging ability.

The in vitro free radical scavenging assays are simple, rapid, and can be used for preliminary screening of the antioxidant capacity of natural compounds (Bajpai et al. 2018; Zhang et al. 2016). Therefore, the antioxidant activities of psoralen and isopsoralen were assessed using DPPH, ABTS, and hydroxyl radical scavenging assays. Figure 2 shows that both psoralen and isopsoralen were effective in scavenging free radicals, and scavenging capacity was below that of the VC group (positive control). The scavenging of DPPH radicals and ABTS radicals was better with isopsoralen than with psoralen, whereas hydroxyl radicals were scavenged better with high concentrations of psoralen than with isopsoralen (Figure 2A–C). Free radical scavenging assays provided preliminary evidence of the antioxidant capacity of psoralen and isopsoralen, and the next cellular experiments will further elucidate the antioxidant activity of psoralen and isopsoralen.

### 3.2 Cytotoxicity of Psoralen, Isopsoralen, or H_2_O_2_ to HEK293T Cells

**FIGURE 3:**
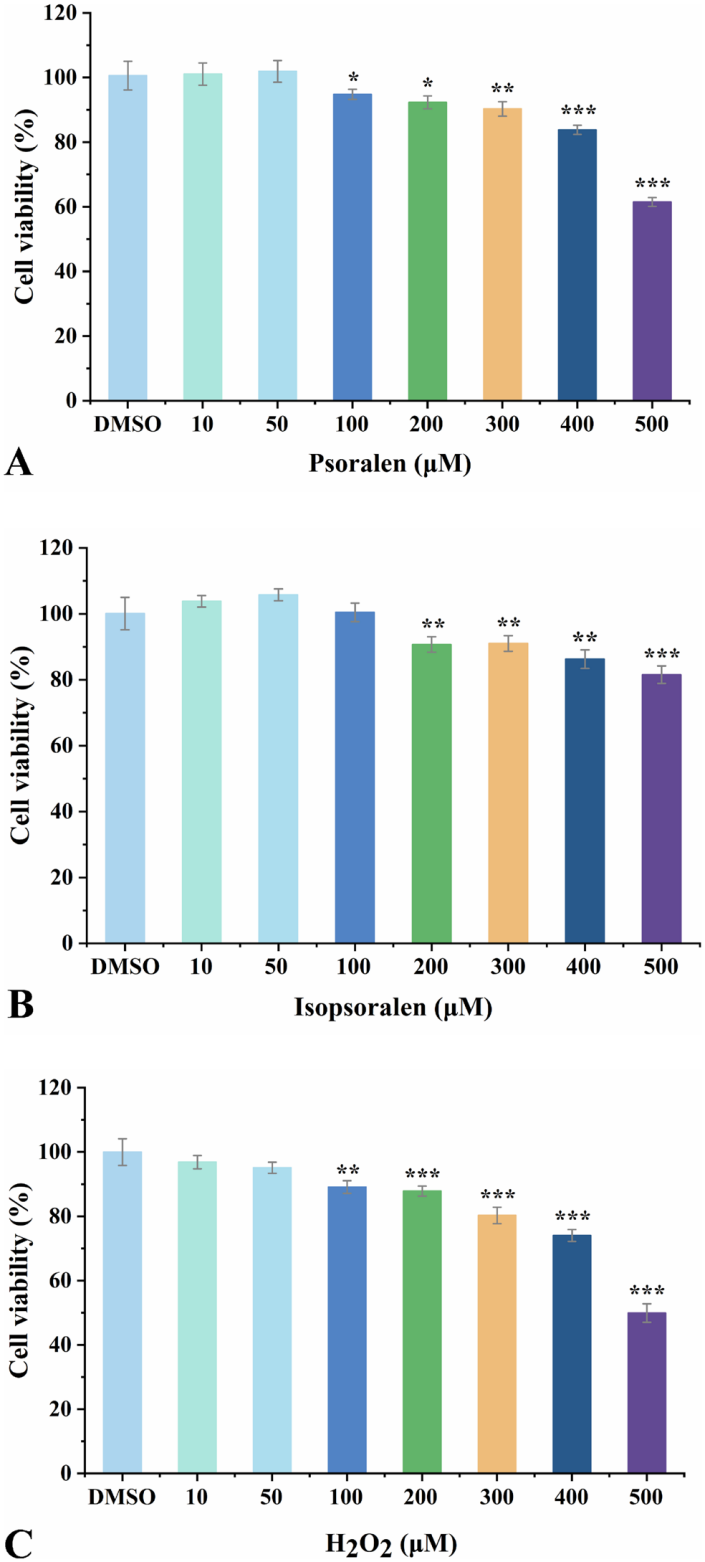
Cytotoxicities of psoralen (A), isopsoralen (B), and H_2_O_2_ (C) on HEK293T cells. Data are expressed as mean ± SD. *p < 0.05, **p < 0.01, and ***p < 0.001 compared with the DMSO groups.

The effects of psoralen, isopsoralen, and H_2_O_2_ on HEK293T cell viability were evaluated using the MTT assay (Figure 3). The DMSO group served as the control group. The viability of HEK293T cells was not significantly influenced by the low concentrations (10–50 μM) of psoralen and isopsoralen. Figure 3A,B shows that the inhibitory effects of psoralen and isopsoralen on HEK293T cells growth were dose‐dependent when the concentrations of psoralen and isopsoralen were higher than 100 μM (*p* < 0.05). Therefore, the concentrations that had no effect on HEK293T cells (10, 20, and 40 μM) were selected for following experiments.

H_2_O_2_ is a strong oxidant that is highly permeable to cell membranes and is commonly used to model oxidative damage to cells (Jin et al. 2015; Li, Zhou, Zheng et al. 2019; Li, Huang, Jie et al. 2019). For this reason, HEK293T cells were stimulated with H_2_O_2_ to establish an oxidative damage model. The growth of HEK293T cells was significantly inhibited at H_2_O_2_ concentrations higher than 100 μM (*p* < 0.01). The half‐inhibitory concentration (IC_50_) of H_2_O_2_ on HEK293T cells was 500 μM, at which the viability was 49.94% (Figure 3C). Hence, follow‐up experiments were performed using 500 μM H_2_O_2_ stimulation for 24 h to establish an oxidative damage model.

### 3.3 Psoralen and Isopsoralen Promote Nrf2 Nuclear Translocation in HEK293T Cells

**FIGURE 4:**
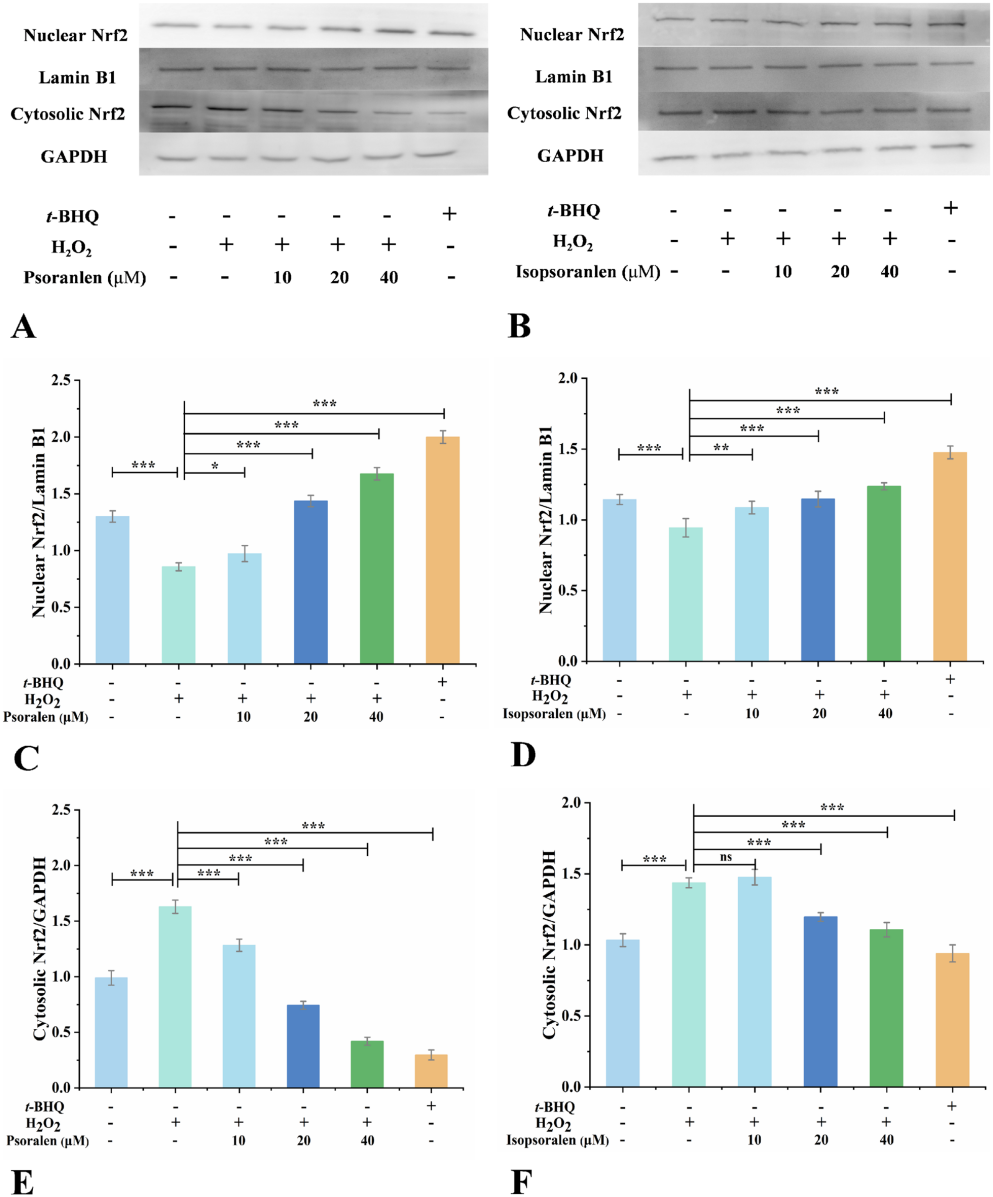
Western blot analysis of the effects of psoralen and isopsoralen on the expression of Nrf2 nuclear and Nrf2 cytoplasmic proteins. Expression levels of nuclear Nrf2 protein and cytoplasmic Nrf2 protein in HEK293T cells treated with (A) psoralen and (B) isopsoralen. The levels of (C) psoralen nuclear Nrf2 and (D) isopsoralen nuclear Nrf2 were quantified by densitometry. The levels of (E) psoralen cytosolic Nrf2and (F) isopsoralen cytosolic Nrf2 were quantified by densitometry. *p < 0.05, **p < 0.01, and ***p < 0.001.

Assessing the essential effect of psoralen and isopsoralen in oxidative defense mechanisms, we investigated whether they induced Nrf2 nuclear translocation in HEK293T cells. The cells were treated with psoralen and isopsoralen, and the Nrf2 protein expression levels in the cytoplasm and nucleus were detected by Western blot. Figure 4 demonstrates that *tert*‐butylhydroquinone at a concentration of 10 μM significantly promoted the expression of Nrf2 nuclear protein (*p* < 0.001). Nrf2 nuclear protein expression was promoted by both psoralen and isopsoralen (Figure 4B,E). It is worth noting that nuclear Nrf2 protein expression was greatest at 40 μM and cytoplasmic Nrf2 protein expression was greatest at 10 μM for both psoralen and isopsoralen, and then it started to decline (Figure 4C,F). The results demonstrated that both psoralen and isopsoralen promoted the nuclear translocation of Nrf2 in HEK293T cells. Nrf2, a key transcription factor that initiates antioxidant defense mechanisms, was released by the Keap1 complex after stimulation with psoralen and isopsoralen, translocated from the cytoplasm into the nucleus, and activated the transcription of downstream antioxidant protein genes (Ai et al. 2022; Furfaro et al. 2016; Xu et al. 2022). Psoralen promotes Nrf2 protein expression and Nrf2 nuclear translocation in murine Spermatogonial stem cells (SSCs) and attenuates radiation‐induced bone damage (Yin et al. 2022). Psoralen promotes translocation of Nrf2 from the cytoplasm to the nucleus, which is consistent with the results of the present study.

### 3.4 ARE Luciferase Activity of Psoralen and Isopsoralen

**FIGURE 5:**
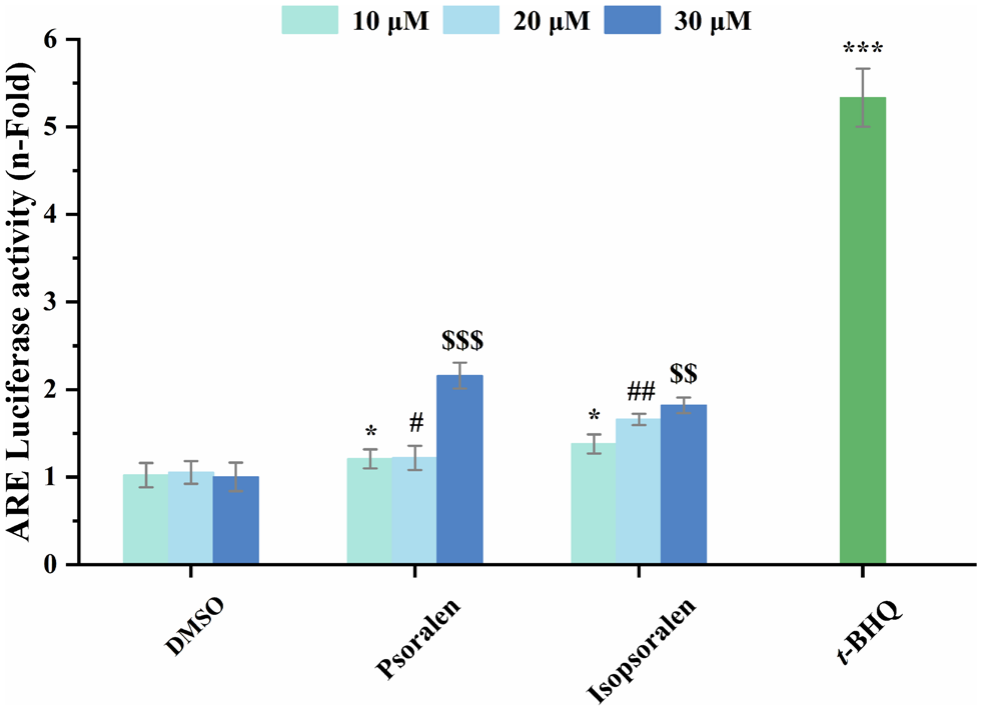
ARE luciferase activity of psoralen and isopsoralen was measured by the luciferase reporter assay. DMSO and *tert*‐butylhydroquinone (*t*‐BHQ) (10 μM) were used as negative and positive controls. ^*/#^, ^##/$$^, and ^***/$$$^ represent statistically significant differences of *p* < 0.05, *p* < 0.01, and *p* < 0.0001, respectively.

As the ARE can regulate antioxidant gene expression and protect cells from endogenous oxidative damage, we investigated the potential effect of psoralen and isopsoralen on ARE luciferase activity. A typical strong antioxidant, *tert*‐butylhydroquinone was found to be 5.33‐fold active against ARE transcription at a concentration of 10 μM. It indicates that the ARE luciferase reporter gene system was successfully established and can be used to assess the effects of psoralen and isopsoralen on ARE activation. As shown in Figure 5, both psoralen and isopsoralen exhibited transcriptional activity against ARE at the concentrations tested (10, 20, and 40 μM). At 40 μM, the transcriptional activity of psoralen on ARE was higher than that of isopsoralen. From these results, both psoralen and isopsoralen enhanced ARE‐driven luciferase activity, suggesting that they are potential Nrf2 activators. ARE is a promoter that regulates cytoprotective genes (Gong et al. 2017; Liang et al. 2019). When the ARE is transcriptionally activated, it activates the expression of genes encoding antioxidant proteins and phase II detoxification enzymes, and protects cells from oxidative damage (Peng et al. 2015; Raghunath et al. 2018).

### 3.5 Psoralen and Isopsoralen Induced the Gene Expression of GCLM, HO‐1, and NQO1

**FIGURE 6:**
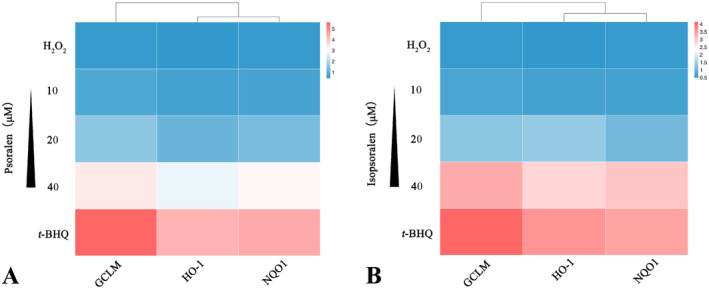
Real‐time quantitative PCR analysis of the effects of psoralen (A) and isopsoralen (B) on *GCLM*, *HO‐1*, and *NQO1* gene expression. The color scale represents relative mRNA levels.

To validate the protective effects of psoralen and isopsoralen against oxidative damage in HEK293T cells, we examined the mRNA expression levels of *GCLM*, *HO‐1*, and *NQO1* using RT‐qPCR. The mRNA expression levels of *GCLM*, *HO‐1*, and *NQO1* genes were decreased after H_2_O_2_ stimulation (Figure 6). Psoralen and isopsoralen induced mRNA expression of all three genes with respect to the H_2_O_2_ group (Figure 6A,B). *GCLM*, *HO‐1*, and *NQO1* are antioxidant genes regulated by Nrf2 and play a central role in protecting cells from oxidative damage (Ji et al. 2021; Liu et al. 2017). Upon cellular exposure to oxidants, Nrf2 nuclear translocation interacts with the ARE, which enhances the expression of antioxidant genes and ultimately protects cells from damage (Shaw and Chattopadhyay 2020; Yao et al. 2014).

### 3.6 Psoralen and Isopsoralen Induced the Protein Expression of GCLM, HO‐1, and NQO1

**FIGURE 7:**
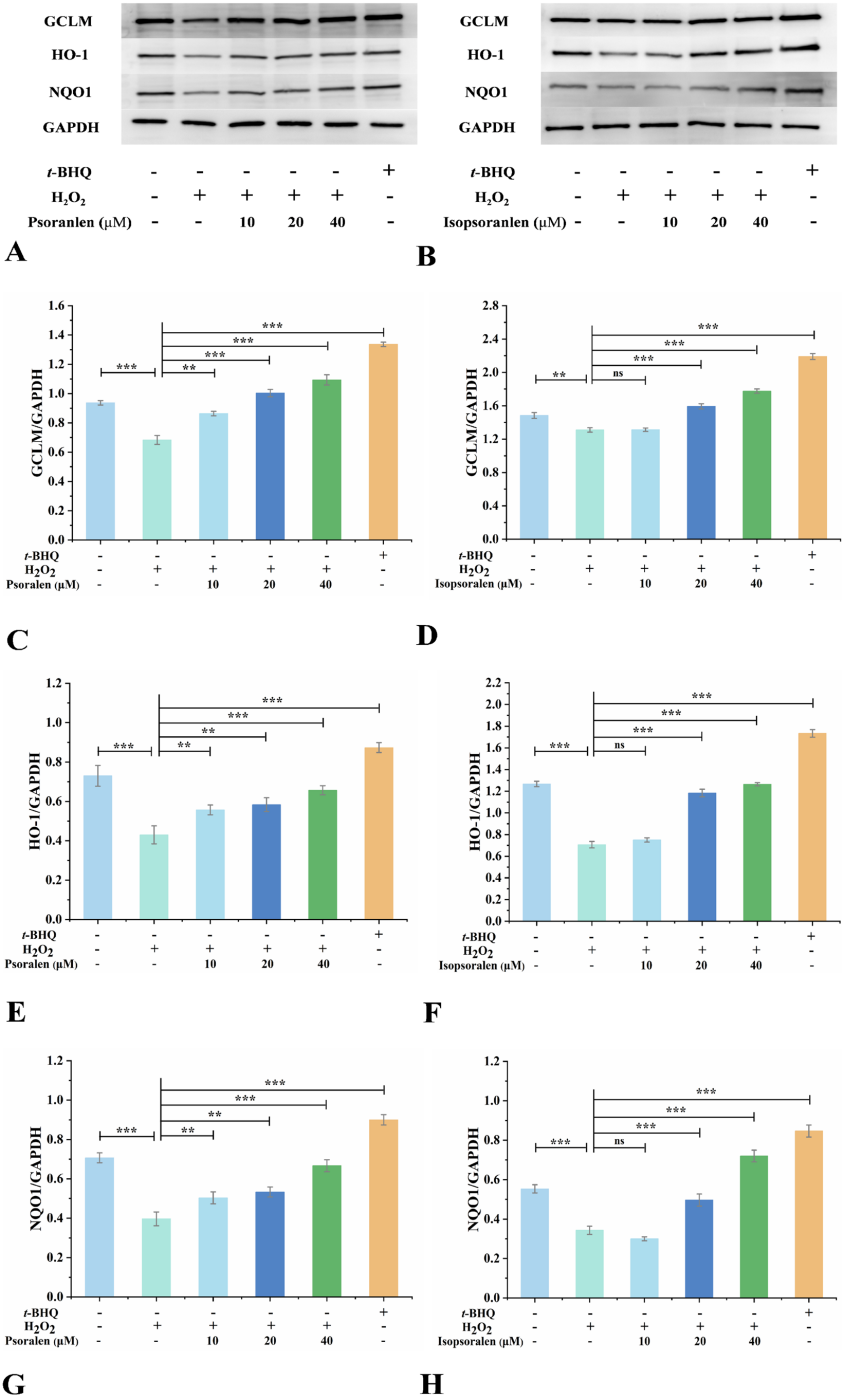
Western blotting analysis of the effects of psoralen and isopsoralen on the expression of GCLM, HO‐1, and NQO1 proteins. Expression levels of GCLM, HO‐1, and NQO1 proteins in HEK293T cells treated with (A) psoralen and (B) isopsoralen. (C) The levels of GCLM by (C) psoralen and (D) isopsoralen were quantified by densitometry. The levels of HO‐1 by (E) psoralen and (F) isopsoralen were quantified by densitometry. The levels of NQO1 by (G) psoralen and (H) isopsoralen were quantified by densitometry. **p < 0.01 and ***p < 0.001.

To further validate the antioxidant capacity of psoralen and isopsoralen, we examined GCLM, HO‐1, and NQO1 protein expression levels using Western blot. The cells were separately treated with psoralen or isopsoralen at concentrations of 10, 20, and 40 μM for 18 h. The treated cells were incubated in 500 μM H_2_O_2_ for 8 h. The results are shown in Figure 7. Upon H_2_O_2_ stimulation, the expression of three Nrf2‐regulated antioxidant proteins was suppressed significantly; in contrast, *tert*‐butylhydroquinone remarkably increased the expression of all proteins (Figure 7A,B). As expected, psoralen and isopsoralen significantly enhanced the expression of antioxidant proteins compared to the H_2_O_2_ group (Figure 7C–H). Consistent with the RT‐qPCR results, both psoralen and isopsoralen activate the Nrf2‐ARE pathway. In summary, the present study demonstrated that psoralen and isopsoralen protected HEK293T cells from H_2_O_2_‐induced oxidative damage and played an antioxidant role in maintaining cellular homeostasis.

### 3.7 Binding Mode of Keap1 With Psoralen and Isopsoralen

**FIGURE 8:**
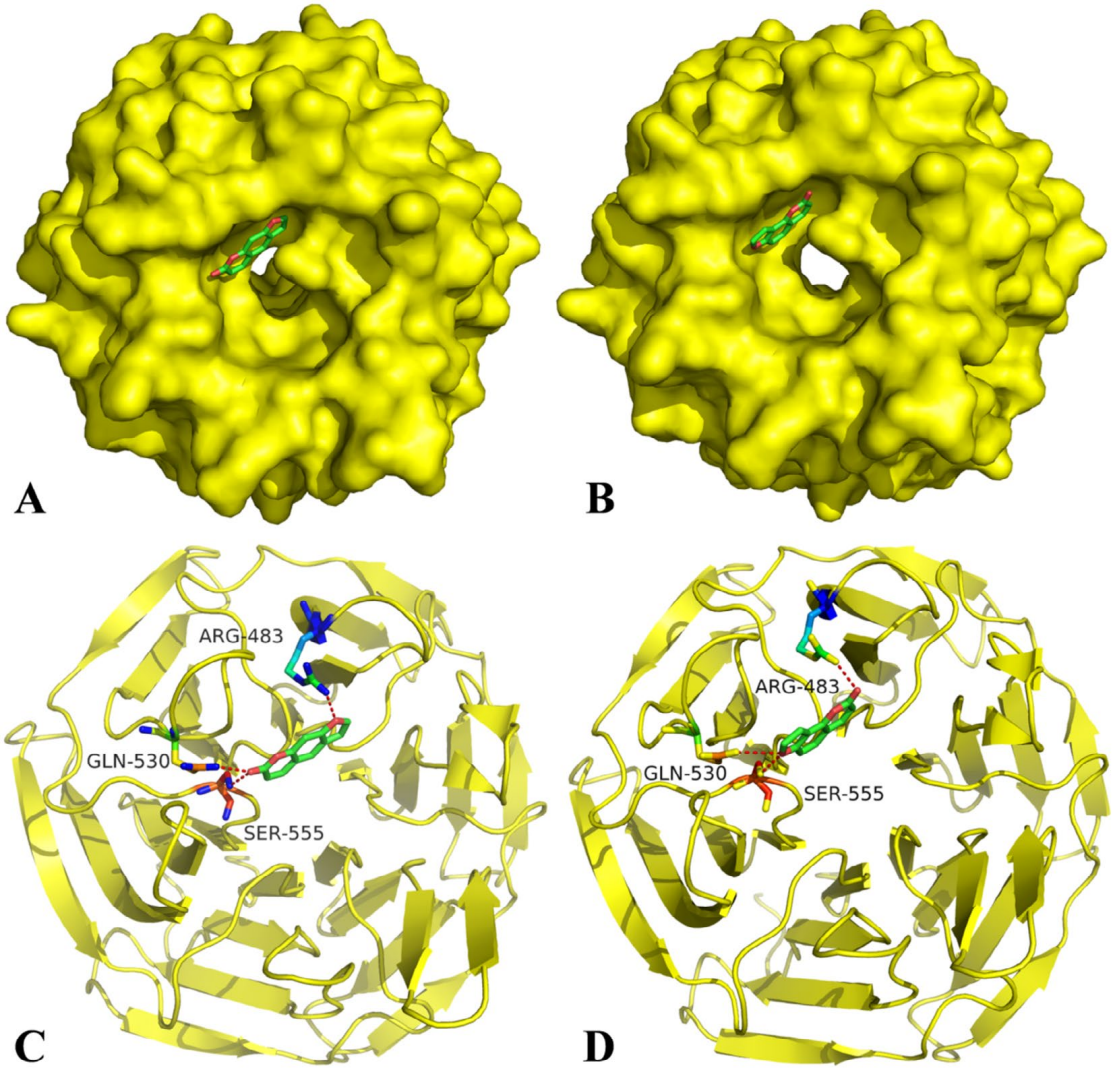
The binding mode of psoralen (A, C) and isopsoralen (B, D) in the binding pocket of the Keap1 Kelch domain. Hydrogen bonds are shown as red dotted lines.

The results of molecular docking showed that both psoralen (Figure 8A) and isopsoralen (Figure 8B) were located in the top of the central pocket of the Keap1 Kelch domain. As shown in Figure 8C, psoralen formed hydrogen bonds with Arg483, Gln530, and Ser555, contributing to its binding in the central pocket of Keap1. Similarly, isopsoralen also formed hydrogen bonds with these three amino acid residues (Figure 8D). Psoralen and isopsoralen play a key functional role in the formation of protein–ligand complexes through hydrogen‐bonding interactions. In addition, psoralen (Figure 8A) and isopsoralen (Figure 8B) could form hydrophobic interactions with the hydrophobic binding pocket, which also helped stabilize the protein–ligand complexes. Collectively, these findings suggest that both hydrogen‐bonding interactions and hydrophobic interactions were conducive to the stability of these two complexes. The binding free energies of psoralen and isopsoralen with Keap1 were − 6.13 and − 6.34 kcal/mol, respectively, suggesting that these two furocoumarins were natural ligands of Keap1.

## 4 Conclusions

This work investigated the activation of Nrf2 by psoralen and isopsoralen, as well as the binding interaction of Keap1 with these two furocoumarins. Psoralen and isopsoralen exhibit enhanced free radical scavenging ability. The reporter gene showed that both psoralen and isopsoralen activate ARE luciferase activity. RT‐qPCR and Western blot showed that psoralen and isopsoralen upregulated the mRNA expression levels of *HO‐1*, *NQO1*, and *GCLM*, while promoting the protein expression levels as well. Both psoralen and isopsoralen were located in the top of the central pocket of the Keap1 Kelch domain, suggesting that they are natural ligands of Keap1. In conclusion, both psoralen and isopsoralen activate Nrf2 through interaction with Keap1, thereby serving as natural antioxidants.

## Author Contributions

**Chengyu Lv:** investigation, data curation, writing – original draft. **Song Wang and Jing Liu:** methodology, data curation. **Yihao Chen and Chang Sun:** original draf. **Chao Wang:** formal analysis. **Tiezhu Li, Cuiping Yuan and Fengxian Qin:** funding acquisition, writing – review and editing.

## Ethics Statement

This article does not include any human or animal experiments.

## Conflicts of Interest

The authors declare no conflicts of interest.

## Data Availability

Data will be made available based on request.
